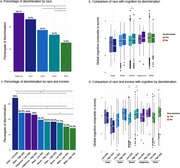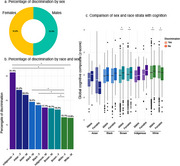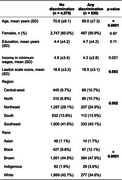# Intersectional impact on cognition by lived discrimination: an ELSI‐Brazil study

**DOI:** 10.1002/alz70860_104065

**Published:** 2025-12-23

**Authors:** Wyllians Vendramini Borelli, Joana Emilia Senger, Lucas Uglione Da Ros, C. Elizabeth Shaaban, Eduardo R. Zimmer

**Affiliations:** ^1^ Universidade Federal do Rio Grande do Sul, Porto Alegre, Rio Grande do Sul, Brazil; ^2^ Universidade Federal do Rio Grande do Sul, Porto Alegre, RS, Brazil; ^3^ Universidade Federal Do Rio Grade Do Sul, Porto Alegre, Rio Grande do Sul, Brazil; ^4^ University of Pittsburgh Alzheimer's Disease Research Center (ADRC), Pittsburgh, PA, USA; ^5^ Federal University of Rio Grande do Sul (UFRGS), Porto Alegre, RS, Brazil; ^6^ Brain Institute of Rio Grande do Sul (InsCer), PUCRS, Porto Alegre, Rio Grande do Sul, Brazil; ^7^ McGill Centre for Studies in Aging, Montreal, QC, Canada

## Abstract

**Background:**

Intersectionality provides a framework for understanding how structural and individual identity factors influence cognition in older adults, highlighting the overlap of multiple reinforcing systems of oppression. In this study, we examined the impact of lived experiences of discrimination on cognitive scores across various intersectional identity strata.

**Method:**

Data from the first wave (2015–2016) of the Brazilian Longitudinal Study of Aging (ELSI‐Brazil), a large nationwide dataset, were used for this study. Clinical, cognitive, and demographic data were collected from older adults. Discrimination was assessed through five questions addressing experiences of discrimination. A global cognitive composite score was calculated based on orientation, fluency, and immediate and delayed memory recall. Functional ability was measured using the modified Lawton and Brody scale. Stratified linear regression models were employed to identify predictors of cognition and functional ability, adjusting for age, sex, education, and socioeconomic status (SES).

**Result:**

Out of 5,409 older adults with complete responses regarding discrimination, 830 (15.3%) reported experiencing discrimination (Table 1). The frequency of reported lived discrimination varied significantly across racial groups, with Indigenous individuals exhibiting the highest rates (26.1%, *p* < 0.001), followed by Black individuals (18.5%, *p* < 0.001, Figure 1a). Overall, reported lived discrimination was not associated with cognitive scores (*p* = 0.56). However, discrimination was a significant predictor of cognition in Brown individuals (β = ‐0.08, *p* = 0.01), but not in other racial groups (*p* > 0.05, Figure 1b). When stratified by income and race (Figure 1c), the effect of discrimination on cognition was more pronounced in individuals with low SES (Figure 1d). Regarding sex and race (Figure 2a), Indigenous women reported the highest levels of discrimination, while White women reported the lowest (Figure 2b). Additionally, sex was associated with cognition in Brown and White individuals, but in opposite directions (Figure 2c).

**Conclusion:**

Discrimination has an important impact on cognitive scores of older adults, varying according to race, sex and income. Importantly, indigenous, and brown individuals, as well as women and low SES were impacted differently by discrimination. These relationships should be further explored to target public policies against more vulnerable intersectional groups.